# Exosomes: Origins and Therapeutic Potential for Neurodegenerative Disease

**DOI:** 10.3389/fnins.2017.00082

**Published:** 2017-02-27

**Authors:** Diana K. Sarko, Cindy E. McKinney

**Affiliations:** ^1^Department of Anatomy, Southern Illinois University School of MedicineCarbondale, IL, USA; ^2^Department of Genetics and iPSC Stem Cell Lab, Edward Via College of Osteopathic MedicineSpartanburg, SC, USA

**Keywords:** exosomes, biogenesis, neurodegeneration, nanotherapeutics

## Abstract

Exosomes, small lipid bilayer vesicles, are part of the transportable cell secretome that can be taken up by nearby recipient cells or can travel through the bloodstream to cells in distant organs. Selected cellular cytoplasm containing proteins, RNAs, and other macromolecules is packaged into secreted exosomes. This cargo has the potential to affect cellular function in either healthy or pathological ways. Exosomal content has been increasingly shown to assist in promoting pathways of neurodegeneration such as β-amyloid peptide (Aβ) accumulation forming amyloid plaques in the brains of patients with Alzheimer's disease, and pathological aggregates of proteins containing α-synuclein in Parkinson's disease transferred to the central nervous system via exosomes. In attempting to address such debilitating neuropathologies, one promising utility of exosomes lies in the development of methodology to use exosomes as natural delivery vehicles for therapeutics. Because exosomes are capable of penetrating the blood-brain barrier, they can be strategically engineered to carry drugs or other treatments, and possess a suitable half-life and stability for this purpose. Overall, analyses of the roles that exosomes play between diverse cellular sites will refine our understanding of how cells communicate. This mini-review introduces the origin and biogenesis of exosomes, their roles in neurodegenerative processes in the central nervous system, and their potential utility to deliver therapeutic drugs to cellular sites.

## Exosome biogenesis

Exosomes were first reported (Pan and Johnstone, 1983) as a way red blood cells (RBCs) released excess cell membrane. It has since been realized that exosomes, defined as extracellular vesicles approximately 30–120 nm diameter size, are another member of the cellular secretome, acting as both a paracrine messenger affecting adjacent cells and a systemic messenger circulating in the blood. Exosomes develop from the in-budding of endosomes, which in turn forms multi-vesicular bodies (MVBs) that contain intra-luminal vesicles (ILVs) (Cocucci and Meldolesi, 2015). The MVBs then fuse with the cell membrane of various cell types to release ILVs extracellularly as exosomes, which then serve as messengers communicating with other cells. Through such intercellular communication—including penetration of the blood brain barrier—exosomes represent optimal targets to harness as a drug delivery vehicle of therapeutic cargo (Stremersch et al., 2016).

**Figure 1:**
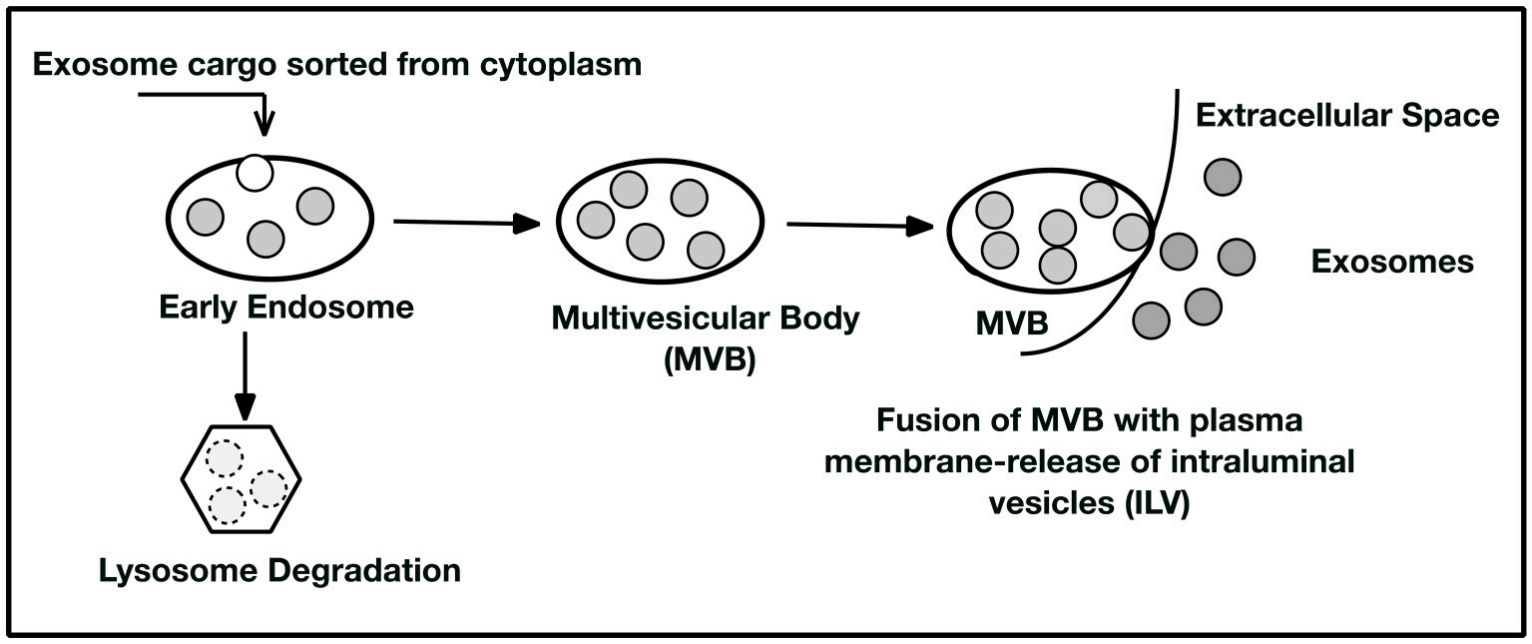
**Exosome Biogenesis**. Cytoplasmic cargo is selected by sorting mechanisms (as yet not comprehensively elucidated) and placed into early endosomes. The endosomes may then enter the lysosomal degradation pathway where the exosome contents are degraded and recycled for cellular use or removed as debris. In the second pathway, the endosome forms a multivesicular body (MVB) containing many intraluminal vesicles (ILVs). The MVB can deliver the ILV to the plasma membrane where they are extruded and become exosomes. These exosomes may act as cell to cell messengers to nearby recipient cells or be picked up in the circulation from the extracellular space. Consequently, exosomes may affect distant cellular sites by being captured at their plasma membranes by receptor-ligand interactions. See references (Keller et al., 2006; Trajkovic et al., 2008; Colombo et al., 2014; Kowal et al., 2014; Hurley, 2015) for further detail.

Exosomes carry intracellularly sorted cargo that is encapsulated in a lipid bilayer bound vesicle. The MVBs carry ILVs to the cytoplasmic side of the plasma membrane where the MVB fuses and releases the ILVs as exosomes to the extracellular space near recipient cells. Alternatively, the endosome can fuse with a lysosome where the contents of the MVB are degraded and recycled within the cell (Figure 1). The exosome's internal cargo reflects the status of the cell's health and/or disease and may affect nearby cells by binding to their membranes and extruding their cargo (Eitan et al., 2016). A growing body of evidence suggests that exosomes can influence both cell health and pathology of cells by the content of the vesicle (Pegtel et al., 2014). Investigators are also exploring the use of these nanoparticles as natural therapeutic delivery vehicles. They can travel systemically, can pass through the blood-brain barrier (Li et al., 2017), can escape stimulating immune responses, and, because the lipid vesicle protects the exosomal contents, can resist degradation from both endogenous and exogenous enzymes and RNAses.

## Exosome content

**Figure 2:**
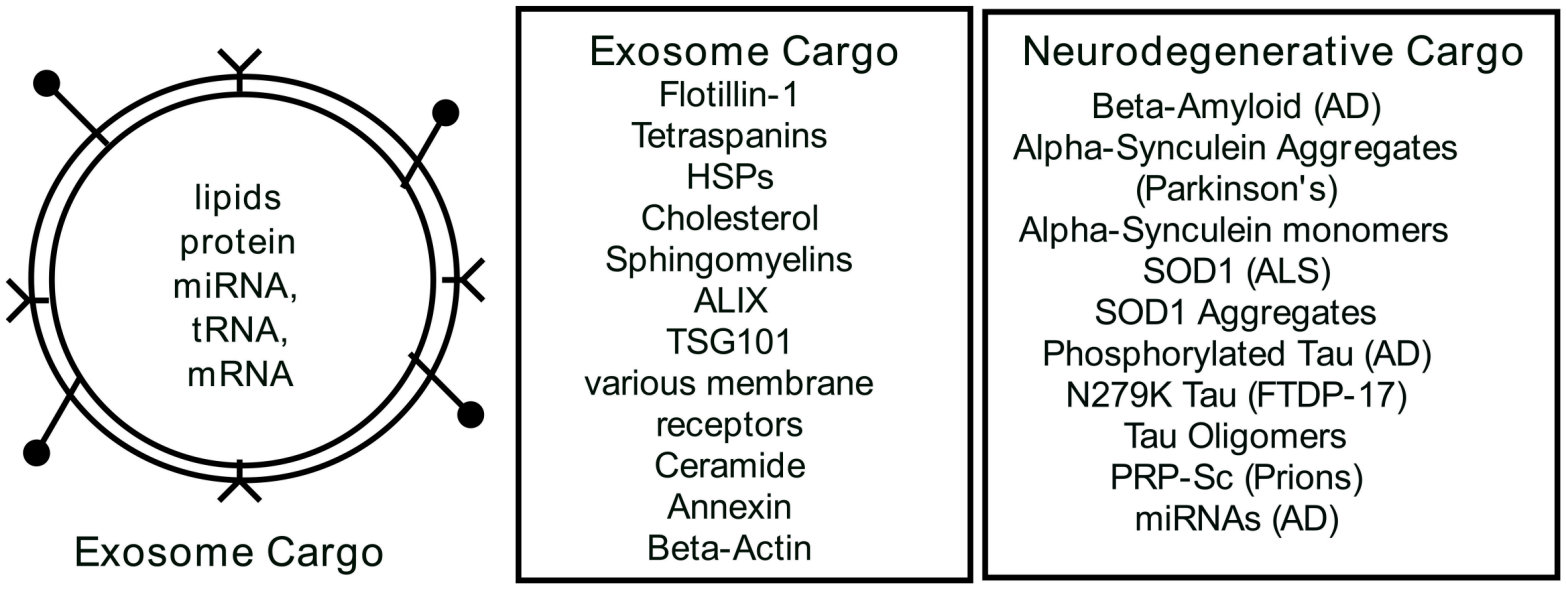
**Exosome Content**. Exosomes are a bilayer lipid membrane that encapsulate cellular cargo selected from the cytoplasm by sorting mechanisms. The lipid membrane may also contain ligands for recipient cell receptors at near or distant systemic sites. Cataloged in exocarta.org is a list of commonly found proteins and RNAs in exosomes. Recent analyses of exosomes from neurodegenerative cells have documented disease-associated cargoes in the exosomes. For further details see references (Bellingham et al., 2012a,b; Taylor and Shah, 2015; Benussi et al., 2016; Willms et al., 2016).

The biological content of exosomes (Figure 2) consists of lipids, proteins and RNAs that can be transferred to recipient cells where they can trigger cellular responses (Lopez-Verrilli et al., 2016; Willms et al., 2016). Exosome content is determined at the budding endosome compartment and requires active sorting by specific proteins of the endosomal sorting complex (ESCRT) to package the cargo. Two proteins from this complex ALIX (ALG2 interacting protein X) and TSG101 (tumor susceptibility gene 101) are commonly found in exosomes and used as marker proteins. Flottilin-1 is also found in exosomes as a marker protein. There also appears to be an ESCRT independent path to exosome cargo sorting (Trajkovic et al., 2008; Villarroya-Beltri et al., 2014; Zhang et al., 2015). A complete database of exosomal proteins can be found at Exocarta (www.exocarta.org) (Keerthikumar et al., 2015) where over 100 proteins are listed as exosomal biomarkers. Exosomes are likely aheterogeneous population of vesicles in which cargo can differ depending on the physiological state of the cell producing the exosomes and the sorting mechanisms invoked for packaging. For example, exosome analysis of vesicle release from cancer cells (Palma et al., 2012) suggested that there were customized particles containing specific miRNAs. Thus, each cell releasing exosomes may be customizing subpopulations of vesicles containing differentially defined cargo (Willms et al., 2016). Exosome content also varies with the cell source of the exosomes. RNA populations in exosomes appear distinct from the cytoplasmic RNA profile suggesting active sorting to the exosomes (Baglio et al., 2015). It is reported that exosomes have specific miRNA profiles, perhaps indicating putative targets of regulation in the recipient cell. Baglio et al. (2015), reported that five miRNAs accounted for 50% of the exosomal content of bone marrow derived mesenchymal stem cells (BMSC) and that these miRNAs function in bone marrow differentiation and proliferation. In addition to miRNA content, there is a substantial collection of tRNA and tRNA halves that may also contribute to cellular regulation in some way. Many investigators have focused on the selective cargo found in exosomes and these continued explorations will inform our understanding of exosome function in the future.

## Exosomes as potential therapy tools

**Figure 3:**
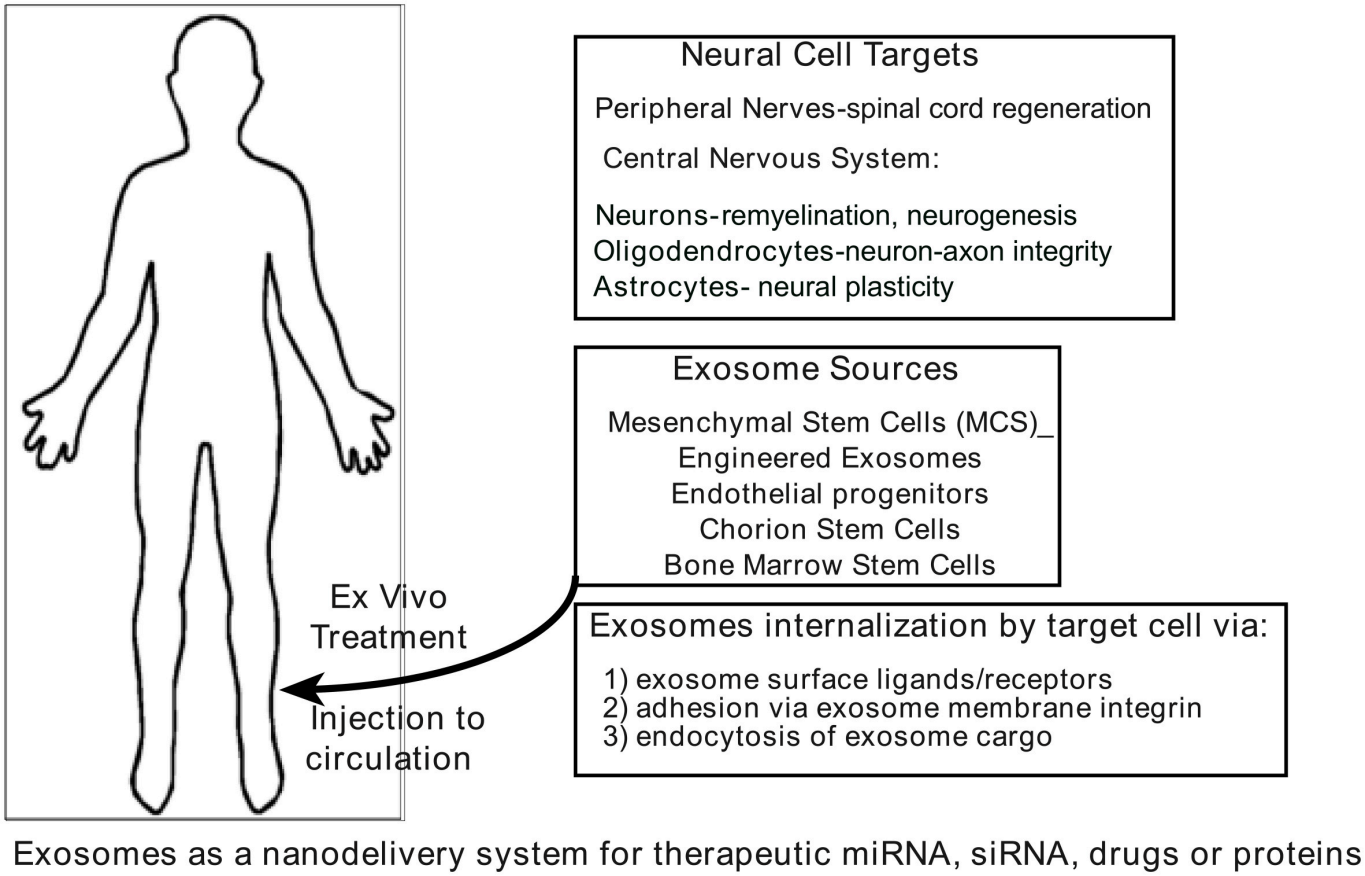
**Exosomes as carriers of therapeutics**. It is proposed that natural or synthetic exosomes could be used as therapeutic tools because the lipid-bound vesicles resist degradation. They are shown to carry and deliver cargo via a secretome from one cell to another. Exosomes have the unusual ability to cross the blood-brain barrier and therefore present a unique opportunity to deliver therapeutics to targeted brain regions. See references (Lamichhane et al., 2015b; Lener et al., 2015; Aryani and Denecke, 2016; Lopez-Verrilli et al., 2016; Yim and Choi, 2016) for further detail.

The ability of exosomes to communicate among cells locally and systemically has prompted evaluation of repurposing these vesicles as carriers of therapy (Figure 3) for a number of diseases such as cardiomyopathies (Loyer et al., 2014; Iaconetti et al., 2016; Ibrahim and Marban, 2016), cancer (Ahadi et al., 2016; Aqil et al., 2016; Choy and Jandial, 2016), and neurodegeneration diseases (Kalani et al., 2014; Bellingham et al., 2015; Kramer-Albers and Hill, 2016). Exosomes from cellular sources (bone marrow, mesenchymal stem cells, and others) or re-engineered exosomes that carry selected cargo (drugs, therapeutic proteins, and others) are able to transport cargo across the blood-brain barrier and deliver it to the brain (Andras and Toborek, 2016). It follows that if the cargo storage and system transport capacity of exosomes can be adapted to carry therapeutic entities to diseased brain regions, exosomes may be used to help alleviate a wide range of neuropathologies. Exosome secretion has been confirmed from different cell sources including neurons (Janas et al., 2016), microglia (Brites and Fernandes, 2015), astrocytes (Verkhratsky et al., 2016), oligodendrocytes (Budnik et al., 2016), and neural stem cells (Sims et al., 2014), and their presence in cerebrospinal fluid has also been reported (Chiasserini et al., 2014). Recent findings suggest that exosomes contain unique biological cargo that represent the physiological health or pathophysiological state of their originating cell source (Kalani et al., 2014). Exosomes have several features that make them accessible as a novel therapeutic strategy for neurological diseases. They are cellular derived membrane bound vesicles that function as biological transporters; they carry unique, sequestered cargoes between adjacent cells and organs; and they can efficiently cross the blood-brain barrier. Caponnetto et al. (2016) and others have shown that exosomes labeled with DiD from a patient's glioma are efficiently taken up by A172 cultured cells. However, they also show that the isolation method (ultracentrifugation or ExoQuick) yields significant exosome size differences that potentially may affect physical uptake or biological function. Another confounding parameter for evaluation of exosomes as an effective nanotherapeutic is the mechanism of exosome uptake into target cells. It is suggested that exosomes enter cells in multiple ways (endocytosis, plasma membrane fusion, macropinocytosis, and others) so each entry method should be evaluated for efficacious delivery and exosomes potentially designed with “zip codes” to utilize the best delivery route. Since exosomes can deliver cargo directly to recipient cells, the capacity to deliver large amounts of cargo to target cells might be leveraged to include therapeutics in engineered exosomes (Armstrong et al., 2017). This cannot efficiently be achieved with soluble factors only.

Exosomes administered *in vivo* are shown to reduce apoptosis, alter inflammation, reduce cancer growth and enhance myocardial viability (Armstrong et al., 2017). Other small synthetic vectors (liposomes) have been considered for therapy approaches. However, the bioavailability and innate biological nature of exosomes makes them very promising vehicles for therapy. For example, catalase is a promising treatment for Parkinson's Disease but the inability to transport it across the blood-brain barrier in drug loaded nanoparticles encountered two problems, nanotoxicity of the formulation and activation and rapid clearance of the particles by phagocytosis (Haney et al., 2015). A formulation of catalase (exoCAT) obtained by drug-loading exosomes reached target neurons and accumulated in these cells in a Parkinson's mouse model (Haney et al., 2015). Much work needs to be done to assess parameters that need to be met for re-engineering exosomes to a therapeutic role but many promising studies are ongoing (see review by Armstrong et al., 2017).

Exosome therapy also offers safety and regulatory advantages (Lamichhane et al., 2015a) over therapeutic *ex-vivo* cell replacement infusions. First, exosomes do not endogenously replicate, as cells would, and thus cannot become metastatic. Second, exosomes are bilipid vesicles that can contain a manufactured cargo and, consequently, can be bioengineered for quality control and scaled up for dose. Therapeutic exosomes might also be derived from the patient's own cells to reduce or eliminate potential immunological complications. In addition to isolating exosomes from the patient's own cells, plant cell sources (Ju et al., 2013) for cargo engineered exosomes has not escaped consideration (Xitong and Xiaorong, 2016).

Yet with all of the apparent advantages that exosome therapy might offer, there remain barriers to be solved before these therapeutic strategies can be widely implemented. The exosome's loading capacity and the half-life of the cargo need to be determined to understand engineered synthetic cargoes for therapeutics. There needs to be a pharmacological consideration of exosome dosage and assessments of their biodistribution when administered systemically. Finally, the kinetics of exosome uptake at the target cell must be determined, as well as whether the therapeutic delivery of exosome cargo requires bioengineered ligands on the vesicle surface to increase delivery success to selected targets. Further development of exosome therapeutic strategies will require continued investigations into exosome biology.

## Exosomes: targeted therapeutic approaches toward neurodegeneration

Cerebral-derived exosomes are demonstrated to carry unique cargoes and to facilitate cross-talk between and among brain regions in coordination with complex brain activities. The exosome's cargo can mediate neuronal protection and development, nerve regeneration and synaptic plasticity (Lopez-Leal and Court, 2016; Lopez-Verrilli et al., 2016; Wei et al., 2016). Also, it is reported that exosomes deliver regulatory elements to neurological injury sites that appear to aid in protein synthesis and tissue regeneration (De Rivero Vaccari et al., 2016; Wei et al., 2016). Conversely, in other instances, they have a role in fostering neurological disease progression. In addressing issues of neurodegeneration, exosomes have been utilized with particular success against such pathologies as stroke, Amyotrophic Lateral Sclerosis (ALS), Alzheimer's disease, dementia, and Parkinson's disease, as detailed below.

Mesenchymal stem cell-derived exosomes are shown to yield tissue-protective effects in stroke models following neural injury resulting from middle cerebral artery occlusion (Xin et al., 2012). Neurite branch number and length was shown to increase significantly following delivery of mesenchymal stem cell-derived exosomes (Yu et al., 2011). This success presumably resulted from exosomal transfer of microRNA 133 b that has been shown to facilitate neuronal recovery following spinal cord injury (Yu et al., 2011).

Exosomes circulating in the blood also appear to have significant effects on immune responses, which in turn carry implications for neurodegenerative and neuroinflammatory disease states. In particular, ALS (also known as Lou Gehrig's disease) is a fatal, progressive, adult-onset neurodegenerative disease that preferentially impacts upper and lower motor neurons in the cerebral cortex and spinal cord, respectively. This neurodegeneration is accompanied by neuroinflammatory reactions including activation of microglia in the central nervous system (Turner et al., 2004; Boillee et al., 2006) and additional involvement of the peripheral immune system (Kuhle et al., 2009; Zondler et al., 2016b). Recent studies have demonstrated impaired pro-inflammatory cytokine secretion following exosomal stimulation in peripheral monocytes from ALS donors (Zondler et al., 2016a). In addition, exosomal TDP-43 (transactive response DNA-binding protein 43 kD; an aggregation-prone protein characteristic in the histopathology of ALS) was shown to increase monocyte activation (Zondler et al., 2016a). Peripheral monocytes are capable of entering the central nervous system in the ALS disease state (Zondler et al., 2016b). Because monocytes demonstrated preferential uptake of exosomal (compared to free) TDP-43 (Zondler et al., 2016a), the collective evidence indicates that exosomes may serve as immune messengers mediating TDP-43 infiltration of the central nervous system. This in turn may contribute toward (and indicate a target for addressing) the pathogenesis of ALS, including increased neuroinflammation and neurodegeneration.

In addition to immune effects, mutations in the Fused in Sarcoma/Translocated in Liposarcoma (FUS/TLS) gene are thought to contribute to ALS pathogenesis. FUS/TLS interacting partners were identified as exosomal components, and FUS/TLS itself was localized to exosomes (Kamelgarn et al., 2016). FUS/TLS secretion from exosomes may contribute to the communication and spread of this mutation. Thus, exosomes represent a viable target in alleviating the disease progression, pathology, and symptomology characterizing ALS.

Recent studies have also demonstrated that exosomes represent viable therapeutic targets against Alzheimer's disease. Alzheimer's disease is characterized by progressive neuronal loss, neurofibrillary tangles, and an overabundance (and lack of degradation) of β-amyloid peptide (Aβ) in the brain forming amyloid plaques (Hickman et al., 2016). In an attempt to address this imbalance of Aβ accumulation, adipose tissue-derived mesenchymal stem cells have been used to secrete exosomes containing neprilysin (Katsuda et al., 2013). This enzyme is capable of degrading Aβ, thereby offering a potential therapeutic tool via exosomal delivery of neprilysin. This in turn might alleviate Aβ accumulation, and ultimately Alzheimer's symptomology. Mouse models also have been utilized in exosome-centered therapies targeting Alzheimer's disease. Electroporation was used to equip dendritic cell-derived exosomes with short interfering RNA (siRNA) for delivery to the brain, crossing the blood- brain barrier (Alvarez-Erviti et al., 2011). Exosome delivery of siRNA resulted in a significant and dose- dependent knockdown of the mRNA and protein for BACE1, a protease that produces N-terminal cleavage of amyloid precursor proteins that lead to Aβ aggregations. Thus, targeted exosomal delivery of siRNA has the potential to cross the blood-brain barrier to reach the brain and generate specific knockdown to alleviate the pathogenesis of Alzheimer's disease.

Frontotemporal dementia (also known as frontotemporal lobar degeneration, or FTLD) is, as the name suggests, a group of disorders that are characterized by atrophy of the frontal and temporal lobes of the brain. FTLD tends to occur in a younger age group than that associated with Alzheimer's disease, typically between the ages of 40–65. Mutations in the progranulin gene (GRN), leading to a loss of functional progranulin proteins associated with exosomes, are a leading cause of FTLD (Baker et al., 2006; Cruts et al., 2006). Null mutations in GRN are shown to significantly reduce the quantity of exosomes released, and the resulting reduction of progranulin in the brain is thought to underlie the neurodegeneration characterizing FTLD cases associated with GRN mutations (Benussi et al., 2016). This GRN-associated alteration of cell-to-cell communication through exosomes offers a therapeutic target in addressing one aspect of the multifaceted underlying causes of dementia.

Parkinson's disease represents an additional pathological target that is amenable to exosome- centered therapeutic approaches. Parkinson's disease is characterized by a variety of pathological hallmarks, including microglia-mediated neuroinflammation, loss of dopaminergic neurons in the substantia nigra of the midbrain and accumulation of Lewy bodies (aggregates of proteins containing α-synuclein). Mutations in the leucine-rich repeat kinase 2 (LRRK2) gene have been linked to inherited Parkinson's disease (Paisan-Ruiz et al., 2004; Zimprich et al., 2004; Gilks et al., 2005; Khan et al., 2005; Nalls et al., 2014). A recent study demonstrated that levels of autophosphorylated Ser(P)-1292 LRRK2 are elevated in urinary exosomes in cases of idiopathic Parkinson's disease (Fraser et al., 2016). This study further correlated cognitive impairment severity and difficulty in performing daily activities with levels of urinary exosome Ser(P)-1292 LRRK2 (Fraser et al., 2016), indicating exosomal delivery of genetic mutations linked to Parkinson's disease and offering a potential therapeutic target with high specificity. The ability to capture the Ser(P)-1292 LRRK2 containing exosomes in the urine also offers an accessible biomarker of this form of Parkinson's disease.

Misfolded proteins have been implicated in a range of neurodegenerative pathologies, using exosomes to spread the misfolded proteins characteristic of each disease and the end result is to worsen pathogenesis. Such misfolded proteins and their associated neuropathologies include: superoxide dismutase 1 protein in ALS, β-amyloid and tau proteins in Alzheimer's disease, and α-synuclein protein in Parkinson's disease (Wu et al., 2016; Quek and Hill, 2017). Exosomes are not only capable of transmitting misfolded proteins associated with disease states, but also of facilitating pathological aggregations of proteins (and subsequent transmission of these aggregates to previously aggregate-free areas of the central nervous system), thereby expediting neurodegeneration (Howitt and Hill, 2016).

## Conclusions

It is now understood that exosomes play a key role in brain health and disease. Over the last several years, investigators have come to appreciate the biogenesis of exosomes, their role as cell- cell transporters and communicators and their function as part of the cell's secretome. These exosomal activities are appreciated to extend the cell's capacity to deliver both beneficial and detrimental molecules across the systemic organism. Due to the exosomes ability to carry diverse cargoes, they are now considered to be ideal vehicles for delivery of therapeutic molecules to previously inaccessible regions of the brain. Continued exosome studies and their mechanisms and utility will optimize novel, targeted therapeutic approaches in addressing a wide range of devastating diseases.

## Author contributions

CM, DS: conceived manuscript topic. CM: researched and wrote exosome introduction and biogenesis. DS: researched and wrote neurodegenerative disease section with emphasis on exosomes role. CM, DS: edited and revised manuscript. CM: conceived figures.

## Funding

This article is supported by internal funding from the Via Research Foundation.

### Conflict of interest statement

The authors declare that the research was conducted in the absence of any commercial or financial relationships that could be construed as a potential conflict of interest.
